# The impact of endurance training on reducing inflammatory markers in the cardiac tissue of methamphetamine-exposed rats

**DOI:** 10.1371/journal.pone.0356103

**Published:** 2026-08-26

**Authors:** Negin Kordi, Ali Saydi, Rezvan Kheirandish, Sajad Karami, Paulo Gentil, Siavash Saghari

**Affiliations:** 1 Department of Exercise Physiology, Faculty of Sport Sciences, Razi University, Kermanshah, Iran; 2 Faculty of Physical Education and Sport Science, Shahid Rajaee Teacher Training University, Tehran, Iran; 3 College of Physical Education and Dance, Federal University of Goiás, Goiania, Brazil; 4 Department of Physical Education and Sports Sciences, Faculty of Humanities and Social Sciences, University of Kurdistan, Sanandaj, Iran; Rutgers: Rutgers The State University of New Jersey, UNITED STATES OF AMERICA

## Abstract

**Background:**

Methamphetamine (METH) consumption has significant harmful effects on the cardiovascular system, which can be mitigated by endurance training (ET). This study aimed to investigate the effect of ET on inflammatory markers in the heart of METH -exposed rats.

**Methods:**

A total of 104 male Wistar rats were allocated into eight groups: 1) CONT; 2) SAL; 3) METH; 4) METH -W; 5) METH ET; 6) METH +ET; 7) METH +ET-W; 8) METH ET-W-ET. METH treatment consisted of administering 5 mg/ kg per day of METH. ET was conducted five days per week on a treadmill at 0° inclinations. Each session lasted 30 min (3 m/min for the first 5 min, 5 m/min for the next 5 min, and 8 m/min for the remaining 20 min). The levels of serum CK and LDH, along with the expression levels of caspase-1, interleukin-1 beta (IL-1β), and interleukin-18 (IL-18) in myocardial tissue were examined.

**Results:**

METH exposure increased the levels of serum CK and LDH and the expression of caspase-1, IL-1β, and IL-18 (P ≤ 0.05). Withdrawal of METH led to a reduction in the expression of these factors (p ≤ 0.05). Moreover, combining METH withdrawal with six weeks of ET further decreased the levels of serum CK and LDH and the expression of caspase-1, IL-1β, and IL-18 (p ≤ 0.05).

**Conclusion:**

Moderate-intensity ET following METH withdrawal reduced METH -induced inflammation in rats, highlighting its potential as a therapeutic strategy for mitigating drug-induced cardiac damage.

## Introduction

Methamphetamine (METH) is a potent psychostimulant and sympathomimetic drug known to significantly affect physiological functions. The intake of METH is linked to augmented alertness, a rise in heart rate, elevated blood pressure and body temperature, along with a decrease in appetite [1,2]. The chronic use of METH is associated with numerous health issues, such as infections, weight loss, malnutrition, kidney and liver damage, respiratory problems, and cardiovascular diseases [3–5]. In rat models with subjected to chronic METH administration, histopathological analyses reveal cardiac abnormalities characterized by myocyte atrophy, lysis, and necrosis, along with inflammation, interstitial edema, fibrosis, and mitochondrial degeneration [6].

Following injury to cardiac myocytes, various biomarkers associated with heart damage are released into the bloodstream. Enzymes such as creatine kinase (CK) and lactate dehydrogenase (LDH) exhibit elevated serum levels, signifying cardiac injury [7]. Studies have indicated that the use of METH is also associated with heightened levels of these biomarkers [8,9].

The consumption of METH can elevate cytokine expression via various signaling pathways, as well as the expression of proinflammatory cytokines [10]. In cardiovascular diseases, the levels of inflammatory markers such as Interleukin-18 (IL-18) and Interleukin-1 beta (IL-1β) are significantly elevated. This increase is associated with the progression of inflammation and tissue damage in the cardiovascular system [11].

Caspase-1 serves as a crucial enzyme within the immune system, significantly influencing the regulation of inflammation and cellular death. This enzyme initiates inflammatory cascades through the activation of IL-1β and IL-18. These cytokines function as primary mediators in inflammatory processes by attracting immune cells to the site of inflammation and facilitating the secretion of additional inflammatory mediators [12]. Therefore, the expression of Caspase-1, IL-1β and IL-18 could be used as inflammatory markers. The modulation of caspase-1 activation and the enzymatic maturation of IL-1β and precursor IL-18 (pro-IL-18) may be attenuated by various antioxidative and anti-inflammatory pharmacological agents, dietary supplements, and exercise training [13].

Exercise may provide cardioprotection through its anti-inflammatory properties [14], reducing cardiac damage, and modulating inflammatory markers [15,16]. Additionally, physical exercise improves heart rate variability, blood pressure, and respiratory function [17,18] and decreases the markers of cardiac inflammatory associated with METH exposure [19,20]. In this context, Kordi et al. (2024) demonstrated that engaging in moderate-intensity aerobic exercise effectively lowered inflammatory markers in the cardiac tissue of rats following METH withdrawal [21].

Thus, endurance training (ET) might have positive effects on METH-induced myocardial inflammatory markers. Given the differential effects of exercise on cellular signaling pathways, it is crucial to investigate whether ET could influence inflammatory markers in the myocardium during METH administration. This study aimed to examine the effects of ET on inflammatory markers in the heart of methamphetamine-exposed rats.

## Materials and methods

### Animals

Based on sample size calculations from the G*Power software v3.1 it was determined that 104 male Wistar rats would allow the study to achieve 80% (β = 0.80) power at 0.05 (α) significance level and an effect size of 0.40. Rats were purchased from the Pasteur Institute of Iran (Research and Production Complex, Karaj, Iran) at adulthood and maintained under standard laboratory conditions. Rats were obtained at 6 weeks of age from the supplier with an average initial body weight of 200 ± 10 g. The rats were housed in solid polycarbonate cages (4–5 males per cage) and were acclimatized at 12 hours of light and 12 hours of dark at 22 ± 2 °C (70 ± 2 °F) and at 55–65% humidity. The rats were fed with standard chow (with ad libitum access) and given unlimited drinking water in 500 mL bottles. The IAEC of Razi University, Kermanshah, granted ethical approval for this study. It was checked that the ethics of animal care and use conformed to caring and use of animals (National Research Council, 8th Edition, 2011), European Convention of the Protection of Vertebrate Animals (Council of Europe No 123, Strasbourg, 1985), and Iranian regulations on animals (Tehran University of Medical Sciences). All procedures were appropriately reviewed in compliance with Tehran University of Medical Sciences, Iran IACUC policies.

Methods of Sacrifice: After confirming complete anesthesia (absence of pedal reflex and stable breathing), exsanguination via the left ventricle was performed, and death was verified by cessation of heartbeat.

Methods of Anesthesia and Analgesia: For intravenous anesthesia induction, a combination of ketamine (80 mg/kg) and xylazine (8 mg/kg) was injected 10:1 into the intraperitoneal space.

This method of anesthesia ensured deep anesthetic levels, as monitored by the absence of both pedal withdrawal and corneal reflexes, prior to invasive interventions. For more prolonged sedation, as in the case of some tissue collection procedures, ketamine in lower doses (20 mg/kg) was given on the need basis to continuously monitored respiratory rate, heart rate, and reflex responses. Analgesia was given post-procedure (where applicable) using buprenorphine (0.05 mg/kg, subcutaneous) every 8–12 hours in the first 24 hours to prevent pain in accordance to the Guide for the Care and Use of Laboratory Animals. All anesthetic and analgesic agents were prepared fresh daily and were administered by trained personnel to guarantee the correct dose. Efforts to Alleviate Suffering: All approaches aimed to alleviate suffering follow the guidelines of the principles of the 3Rs (Replacement, Reduction, Refinement). To reduce stress levels, the rats were offered a one-week acclimatization period before the experiments. The handling sessions conducted by trained personnel were free of any adverse interactions with the animals. The rats were evaluated daily using a standard welfare scoring system for pain and discomfort, and for any behavioral changes such as reduced grooming, lethargy, atypical aggression, and distress. The animals were also provided with nesting materials and chew blocks to promote the performance of species-typical behaviors. While monitoring for signs of overexertion, rats had to be stopped during sessions if signs of exhaustion, such as failing to maintain a pace-sustained trot, were observed. All procedures were conducted under a veterinarian, ensuring all ethics frameworks were addressed. These procedures were also described for the animal’s post-operative monitoring, where the animal’s vital signs and behavior were assessed to ensure there was no distress or altered consciousness. Severely distressed animals (e.g., enduring withdrawal symptoms or METH reactions) were humanely and veterinarian-guided, euthanized early to prevent undue suffering.

### Experimental design

Following a one-week acclimatization and habituation period, the rats were randomly assigned to eight groups (n = 13 per group):

1. **CONT (control group):** Rats were administered METH treatment for a duration of six weeks, followed by a withdrawal period of 21 days, after which they lived without any intervention for an additional six weeks, and subsequently, they were sacrificed.
2. **SAL (saline group):** After receiving a 0.9% saline solution for six weeks, the rats were given a 21-day break before being killed.
3. **METH:** Rats were administered METH treatment for a duration of six weeks and were subsequently sacrificed 12 hours following the last dose.
4. **METH -W (METH then withdrawal):** Rats underwent METH treatment for six weeks, followed by a 21-day withdrawal period, and were sacrificed 24 hours after the withdrawal period.
5. **METH -ET (METH followed by endurance training):** Initially, rats were administered METH for a duration of six weeks, which was succeeded by a six-week period of ET, after which they were sacrificed 24 hours following the final training session.
6. **METH +ET (METH with endurance training):** Rats underwent simultaneous treatment with METH and ET for a duration of six weeks and were euthanized 12 hours following the final training session.
7. **METH +ET-W (METH with endurance training then withdrawal):** Rats underwent METH treatment simultaneously with the ET regimen for a duration of six weeks, followed by a withdrawal period of 21 days, and were euthanized 24 hours after the final day of withdrawal.
8. **METH +ET-W-ET (METH with endurance training, withdrawal, and training):** Rats were administered METH and engaged in a six-week training program simultaneously, followed by a 21-day withdrawal phase. They then continued the training program for an additional six weeks and were sacrificed 24 hours after the final training session.

### METH and saline treatment protocol

METH doses were 5 mg/kg body weight intraperitoneally, in 0.9% saline, daily for six weeks. SAL group got 0.9% saline over six weeks [20].

### METH withdrawal phenomenon

Observations revealed that withdrawal symptoms after METH treatment became apparent 36–72 hours after the last injection, with a notable exacerbation of symptoms at 98 h [22]. It is suggested that cessation of METH use after six weeks of exposure results in withdrawal symptoms for up to 21 days [23]. Symptoms of withdrawal in rodents include tear secretion, diarrhea, pinning, jumping, runny nose, teeth grinding and piloerection [24].

### Endurance training protocol

To aid in acclimatization, the rats were systematically introduced to a controlled endurance activity regimen utilizing an animal treadmill apparatus (Danesh salar, Iran). This process involved sustaining a consistent speed of 5 m/min at a 0% incline for durations of 10, 20, 30, and 40 minutes on the first, second, third, and fourth days, respectively. This preliminary phase lasted one week and encompassed four sessions. After acclimatization, the rats were divided into experimental training groups that participated in a structured ET protocol, which included 30 minutes of treadmill activity per session, performed five times a week over a six-week duration. The training protocol was divided into three distinct phases: an initial warm-up at 3 m/min for 5 minutes, followed by a moderate phase at 5 m/min for the subsequent 5 minutes, and concluding with a phase at 8 m/min for the final 20 minutes, with all phases executed at a 0% incline [25].

### Tissue sample collection and analysis

All experimental procedures involving animals were carried out in strict compliance with ethical standards to ensure the welfare of the animals. Rats were anesthetized in a sterile environment under resting conditions using ketamine (80 mg/kg) and xylazine (8 mg/kg). The heart was subsequently removed from the aortic root. Following washing with deionized water and weighing, a segment of the left ventricle was isolated for the examination of IL-18, IL-1β, and caspase-1 gene expression. The sample was promptly placed in liquid nitrogen and preserved at −80°C. For RNA extraction, approximately 100 mg of left ventricular tissue was homogenized in 1 ml of QIAzol lysis reagent (Qiagen, Germany) and allowed to incubate at room temperature for 5 minutes. cDNA was synthesized using a Genix kit, following the manufacturer's guidelines.

Gene expression was quantified using quantitative polymerase chain reaction (qPCR) with a Corbett 6000 real-time PCR system (Australia) on an Applied Biosystems platform (USA). The thermal cycling program consisted of three distinct stages (Table 1). The initial stage involved a 10‑min incubation at 95 °C to promote DNA strand separation and activate the DNA polymerase enzyme. The second stage comprised a denaturation step at 95 °C for 15 s, followed by primer annealing at 60 °C for 1 min; this cycle was repeated 40 times. The final stage included a melting curve analysis with sequential incubations at 72 °C for 15 s, 60 °C for 30 s, and 95 °C for 18 s.

**Table 1:** The optimized temperature protocol.

Cycles	Duration of cycle	Temperature	
950c	15 minutes	950c	
40	15–30 seconds	950c	Extension
	30 seconds	600c	Denaturation
	30 seconds	720c	Annealing

Real‑time PCR assays were conducted in duplicate using 96‑well plates, with a total reaction volume of 25 µL. Each reaction mixture contained 12.5 µL of SYBR Green master mix with NOROX (Ampliqon, Denmark), 10 pmol (1 µL) of each gene‑specific forward and reverse primer, 20 ng of genomic DNA (5 µL), and nuclease‑free distilled water. SYBR Green I fluorescent dye was used for signal detection.

The optimized temperature protocol in this study after various investigations is as shown in Table 1.

The ΔCT value was calculated using the equation ΔCT = CT(target gene) – CT(GAPDH), with IL‑18, IL‑1β, and caspase‑1 designated as the target genes. Relative gene expression levels were subsequently determined using the 2^–ΔΔCT method. Oligonucleotide primers specific to IL‑18, IL‑1β, caspase‑1, and the housekeeping gene GAPDH were designed based on sequences retrieved from established genomic databases. Real‑time PCR provided the CT and mean CT values for each sample, which were then processed in Excel to generate the corresponding ΔΔCT values. Final gene expression levels were computed according to the 2^–ΔΔCT calculation. The primer sequences used in this study are listed in Table 2.

**Table 2:** qPCR primer sequences.

Name	(5’ – 3’)		Product length
	Forward	Reverse	
IL-18	GGTCACAGCCAGTCCTCTTA	TGACAAAAGAAACCCGCCTG	**nt 124**
IL-1β	TGTCGTTGCTTGTCTCTCCT	GGGATGATGACGACCTGCTA	**nt 192**
Caspase-1	ATGGACCTGACTGAAGCTCC	ACCTGTGCGATCATGTCACT	**nt 114**
GAPDH	CCCCATTTGATGTTAGCGGG	CAAGTTCAACGGCACAGTCA	**nt 102**

Efforts to minimize animal suffering included the use of preemptive anesthesia, close monitoring of vital signs throughout the procedure by trained personnel, and adherence to established protocols designed to reduce pain and distress. Additionally, all procedures were carried out in the presence of a licensed veterinarian or an experienced animal care technician from the Laboratory Animal Science Research Center to ensure that handling was performed gently and with minimal stress. Post-procedural monitoring was conducted to confirm the absence of any signs of distress or residual consciousness.

### Measurement of LDH and CK-MB

Blood samples were collected from the left ventricle of the rats, centrifuged, and stored at −70°C for analysis. LDH and CK-MB were measured using specialized kits and spectrophotometric techniques. High levels of CK-MB were indicative of myocardial damage18. Serum CK and LDH were measured (PARS AzMUN kit, Tehran, Iran) [26]


$$\begin{array}{c} \text{Lactate }+\text{ NAD }+\text{ H }+\text{ Pyruvate }+\text{ NADH }↔\text{ LDH} \end{array}$$

NADH activity leads to the oxidation of the enzyme LDH. The reduction of NAD to NADH in this process is directly proportional and can be assessed photometrically.

### Statistical analysis

The normality of the data distribution was evaluated using the Shapiro-Wilk test. As the data followed a normal distribution, differences between groups were assessed using one-way Analysis of Variance (ANOVA). If the ANOVA indicated significant differences, Tukey's post-hoc test was applied to identify specific group differences. Statistical analyses were performed utilizing Sigma Plot software (version 14.0) with a pre-established significance level of 0.05.

## Results

**Fig 1:**
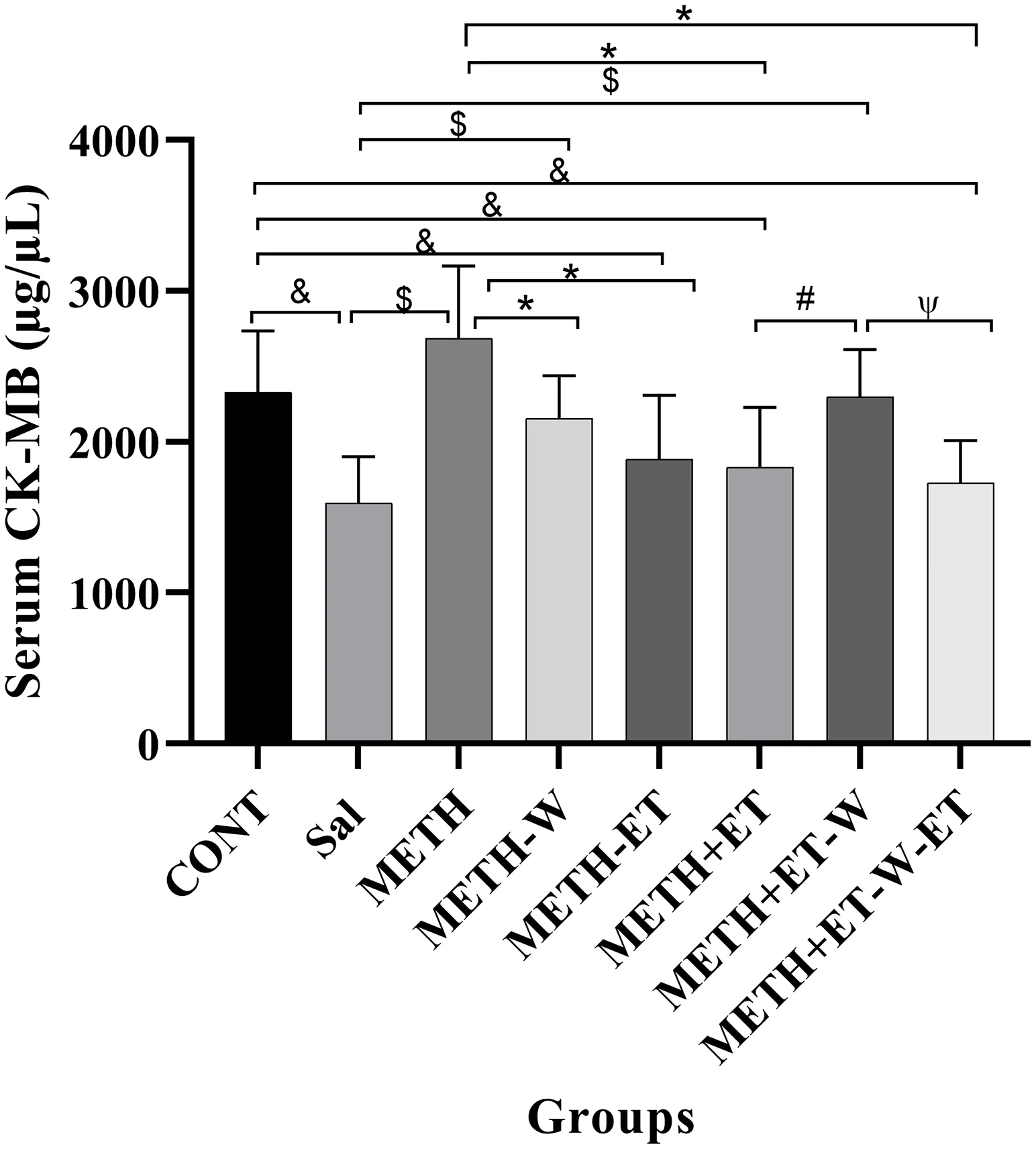
Mean ± standard deviation of serum CK-MB difference between groups. & p < 0.05 vs. CONT group, $ p < 0.05 vs. SAL group, # p < 0.05 vs. METH+ET group, ψ p < 0.05 vs. METH+ET-W-ET group.

**Fig 2:**
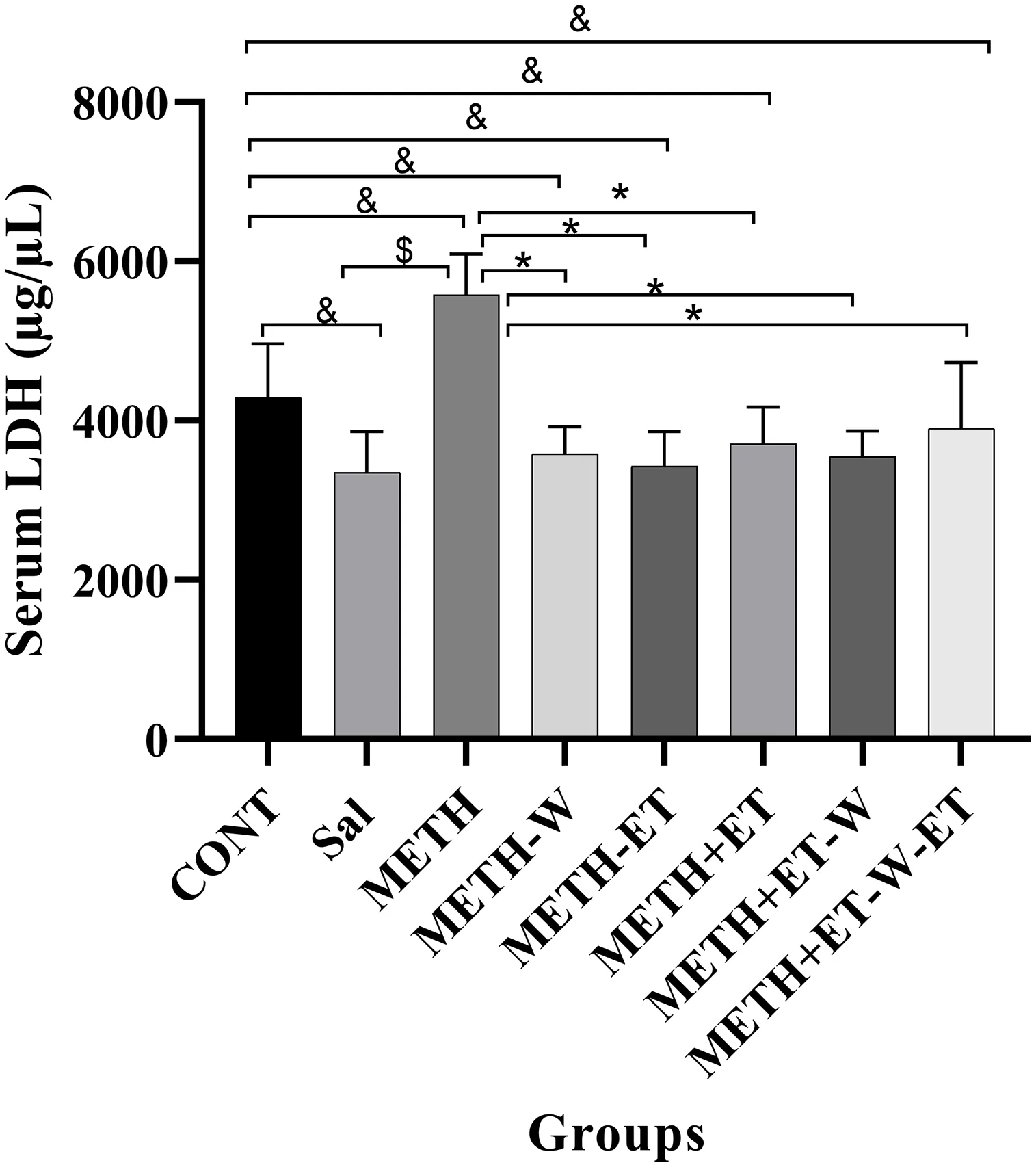
Mean ± standard deviation of serum LDH difference between groups. & p < 0.05 vs. CONT group, $ p < 0.05 vs. SAL group, * p < 0.05 vs. METH group.

The Kolmogorov-Smirnov test showed that the data distribution is normal (P>0/05). The results showed that there is a significant difference in the levels of CK-MB (F = 13.93, P = 0.001) (Fig 1) and LDH (F = 32.68, P = 0.001) between the studied groups (Fig 2).

The post-hoc test results indicate a significant difference in the level of CK-MB. The difference was observed between the CONT group and the Sal group (mean differences = 833.70, p = 0.001), METH-ET group (mean differences = 486.96, p = 0.030), METH+ET group (0.008), and METH+ET-W-ET group (mean differences = 144.44, p = 0.001). Also, a significant difference was observed between the Sal group and the METH group (mean differences = −1145.64, p = 0.001), Sal group and the METH-W group (mean differences = −613.79, p = 0.001), Sal group and the METH+ET-W group (mean differences = −758.35, p = 0.001), METH group and the METH-W group (mean differences = 531.85, p = 0.011), METH group and the METH-ET group (mean differences = 798.90, p = 0.001), METH group and the METH+ET group (mean differences = 754.79, p = 0.001), METH group and the METH+ET-W-ET group (mean differences = 423.94, p = 0.001), METH+ET group and the METH+ET-W group (mean differences = −467.50, p = 0.046), METH+ET-W group and the METH+ET-W-ET group (mean differences = 568.51, p = 0.004).

Regarding LDH, a significant difference was observed between the CONT group and the Sal group (mean differences = 943.87, p = 0.001), the CONT group and the METH group (mean differences = −1285.39, p = 0.001), the CONT group and the METH-W group (mean differences = 705.93, p = 0.005), the CONT group and the METH-ET group (mean differences = 862.84, p = 0.001), the CONT group and the METH+ET group (mean differences = 582.84, p = 0.050), the CONT group and the METH+ET-W-ET group (mean differences = 742.59, p = 0.003), the SAL group and the METH group (mean differences = −229.27, p = 0.001), the METH group and the METH-W group (mean differences = 1991.33, p = 0.001), the METH group and the METH-ET group (mean differences = 2148.24, p = 0.001), the METH group and the METH+ET group (mean differences = 1868.24, p = 0.001), the METH group and the METH-ET-W group (mean differences = 1811.83, p = 0.001), the METH group and the METH-ET-W-ET group (mean differences = 2027.99, p = 0.001).

### IL-18 gene expression

**Fig 3:**
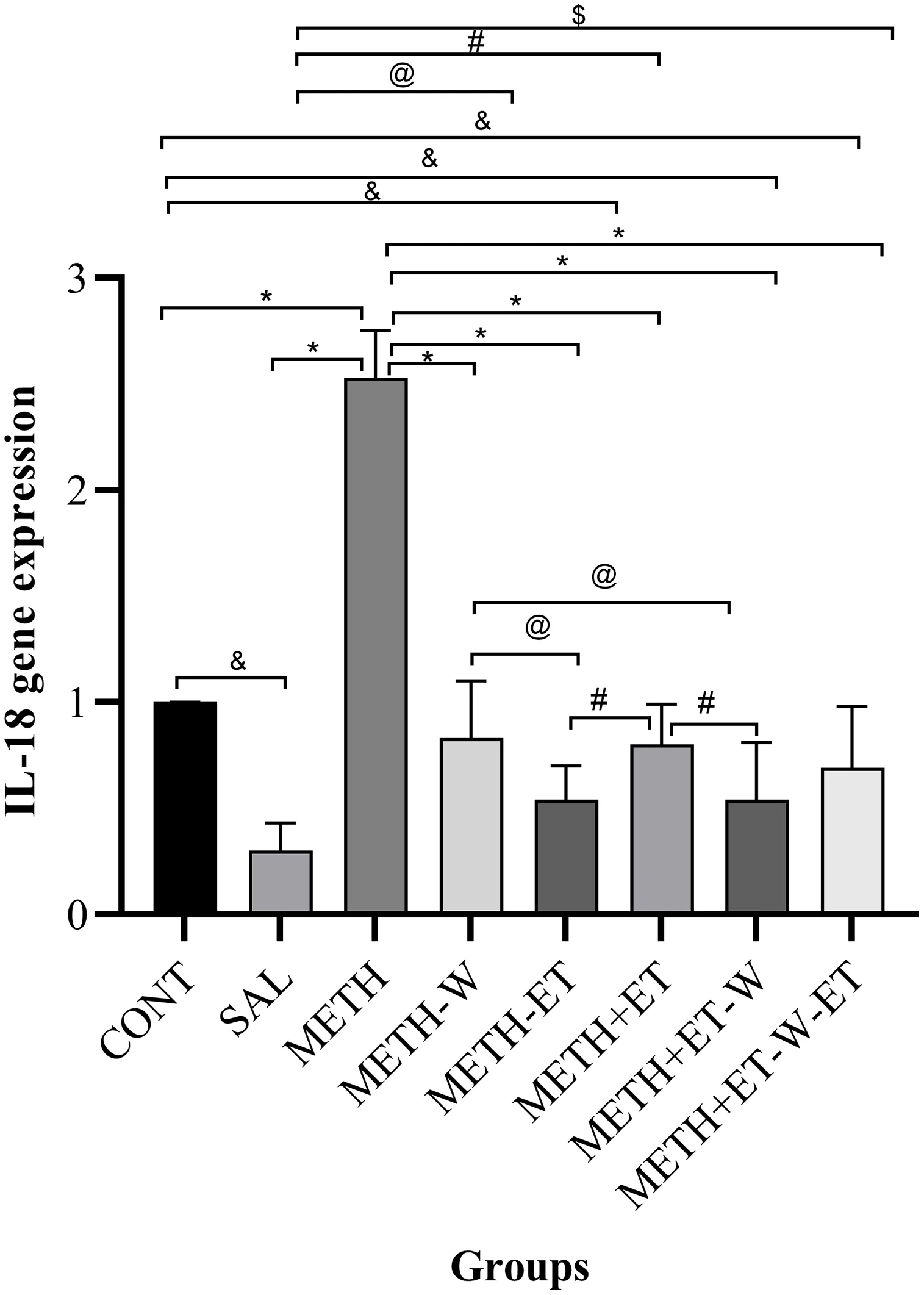
Mean ± standard deviation of IL-18 gene expression relative to GAPDH in the experimental groups. & p < 0.05 vs. CONT group, $ p < 0.05 vs. SAL group, * p < 0.05 vs. METH group, # p < 0.05 vs. METH+ET group, @ p < 0.05 vs. METH-W group.

Fig 3 depicts the analysis of IL-18 expression in cardiac tissues. The ANOVA results revealed a statistically significant difference among the groups (f = 139.24, p = 0.001).

Tukey's post hoc tests showed that IL-18 expression levels were significantly higher in the METH group than in the SAL (mean difference = 2.230, p < 0.001), METH+ET-W (mean difference = 1.990, p < 0.001), METH -ET (mean difference = 1.990, p < 0.001), METH +ET-W-ET (mean difference = 1.840, p < 0.001), METH +ET (mean difference = 1.730, p < 0.001), METH-W (mean difference = 1.700, p < 0.001), and CONT (mean difference = 1.530, p < 0.001) groups. A significant difference was also identified between the CONT and SAL groups (mean difference = 0.700, p < 0.001), between the CONT and METH+ET groups (mean difference = 0.460, p < 0.001), between the CONT and METH-ET groups (mean difference = 0.460, p < 0.001), between the CONT and METH+ET-W-ET groups (mean difference = 0.310, p = 0.007), between the METH-W and SAL groups (mean difference = 0.530, p < 0.001), between the METH+ET-W and SAL groups (mean difference = 0.290, p = 0.016), between the METH-W and METH-ET groups (mean difference = 0.290, p = 0.016), between the METH+ET and SAL groups (mean difference = 0.500, p < 0.001), between the METH+ET and SAL groups (mean difference = 0.260, p = 0.045), between the METH+ET and METH-ET groups (mean difference = 0.260, p = 0.045), and between the METH+ET-W-ET and SAL groups (mean difference = 0.390, p < 0.001) (Fig 3).

### IL-1β gene expression

**Fig 4:**
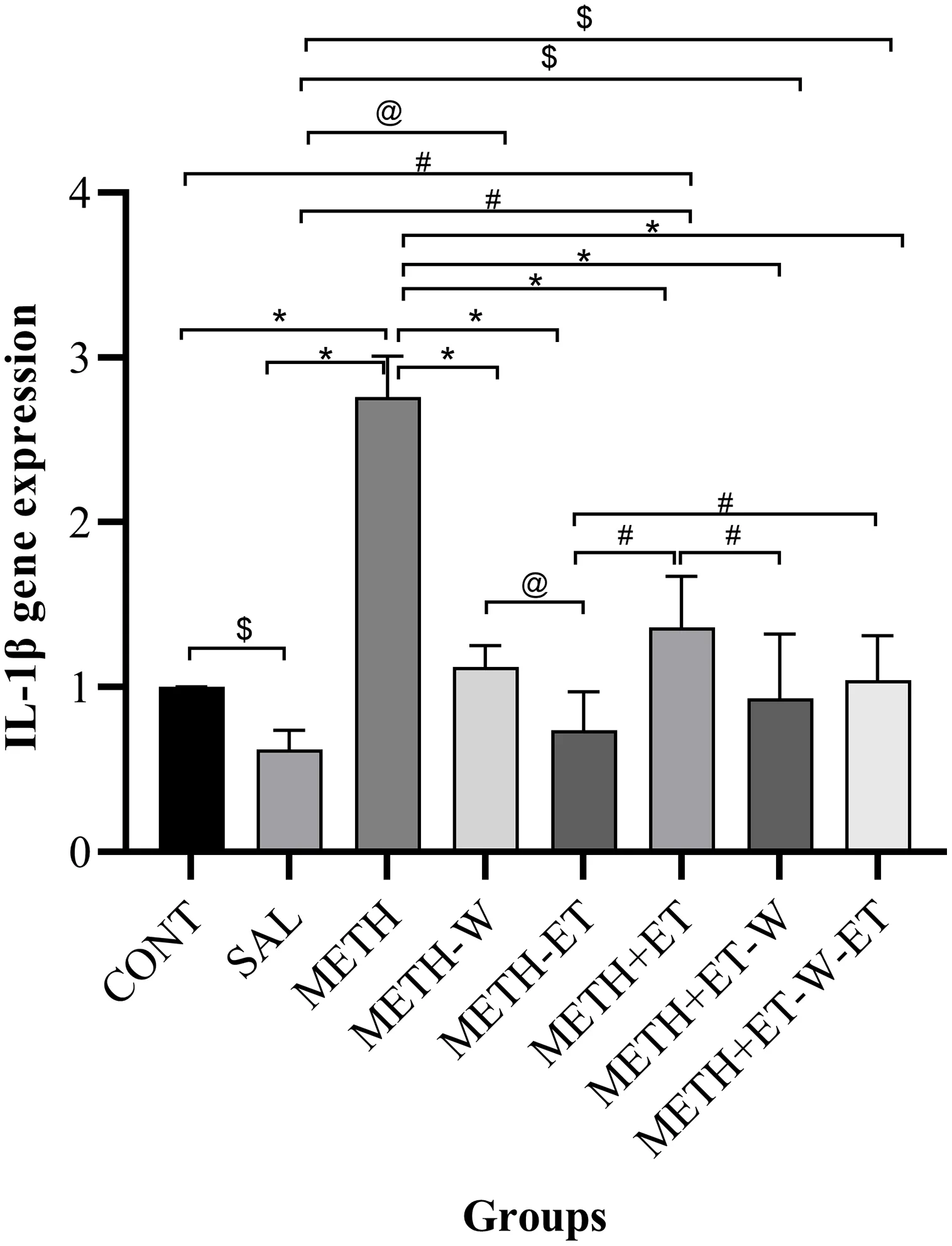
Mean ± standard deviation of IL-1β gene expression relative to GAPDH in the studied groups. $ p < 0.05 versus SAL group, * p < 0.05 versus METH group, # p < 0.05 versus METH+ET group, @ p < 0.05 versus METH-W group.

Fig 4 shows the expression levels of IL-1β in cardiac tissue. One-way ANOVA demonstrated a highly significant difference in IL-1β gene expression among the groups (F = 99.78, p < 0.001).

Tukey's post hoc test further clarified these differences. The METH group showed significantly elevated IL-1β expression compared to the SAL (mean difference = 2.140, p < 0.001), METH-ET (mean difference = 2.010, p < 0.001), and METH+ET-W groups (mean difference = 1.828, p < 0.001). Significant differences were also observed between the METH group and the CONT (mean difference = 1.760, p < 0.001) and METH+ET-W-ET groups (mean difference = 1.720, p < 0.001) as well as between the METH-W (mean difference = 1.640, p < 0.001) and METH+ET groups (mean difference = 1.400, p < 0.001).

Additional comparative analyses revealed significant differences among the other groups. For instance, the M + ET group exhibited significantly higher IL-1β expression than the SAL(mean difference = 0.740, p < 0.001), METH-ET (mean difference = 0.610, p < 0.001), METH+ET-W (mean difference = 0.428, p < 0.001), CONT(mean difference = 0.360, p = 0.006), and M + ET-W-ET (mean difference = 0.320, p = 023) groups did. A significant difference was also identified between the METH-W and SAL groups (mean difference = 0.500, p < 0.001), METH-ET groups (mean difference = 0.370, p = 0.004). Furthermore, a significant difference was also identified between the SAL and METH+ET-W-ET groups (mean difference = 0.420, p < 0.001), CONT groups (mean difference = 0.380, p = 0.003) and METH+ET-W (mean difference = 0.312, p = 0.029) (Fig 4).

### Caspase-1 gene expression

**Fig 5:**
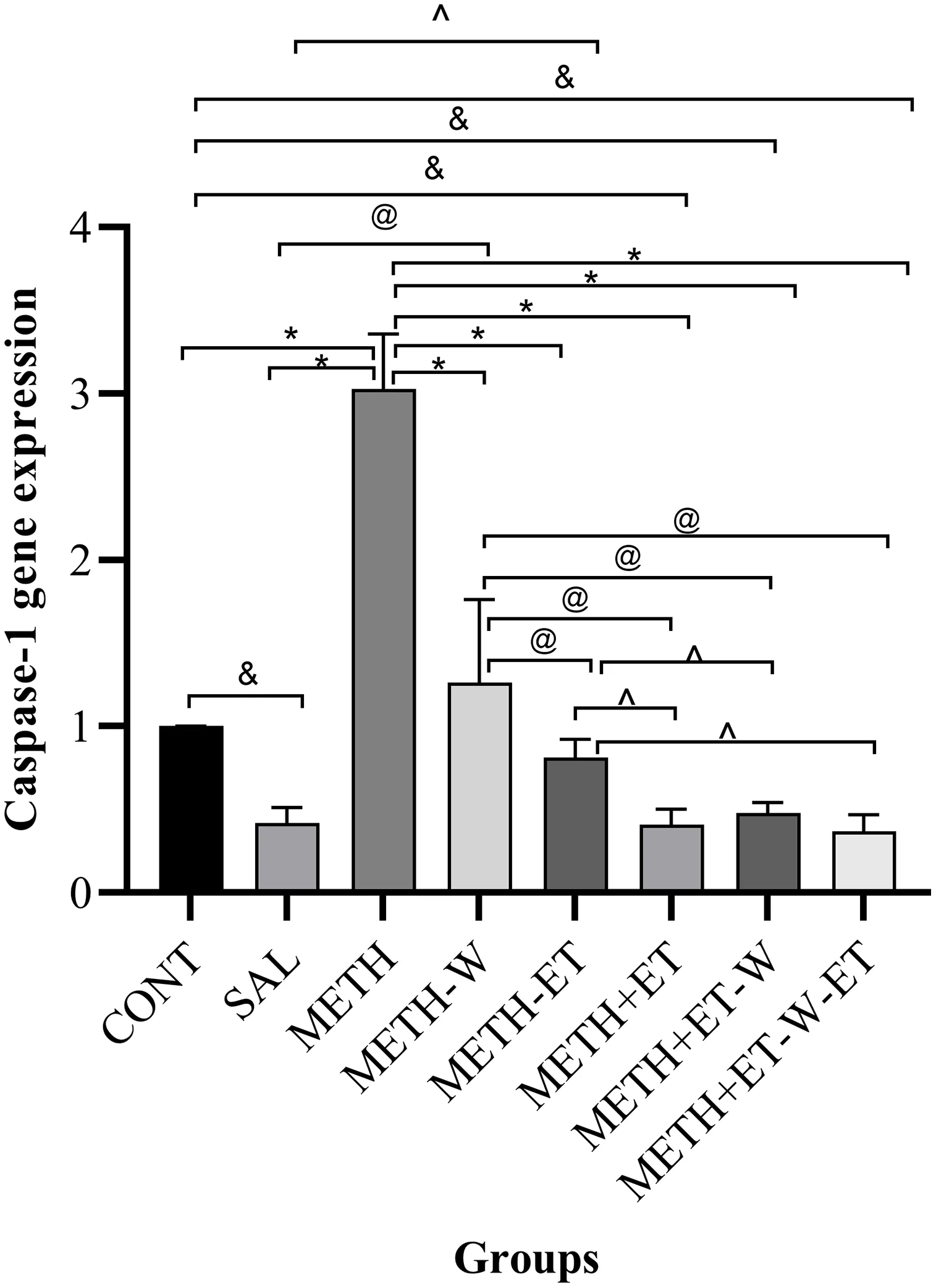
Mean±standard deviation of the relative expression of Caspase-1 gene compared to GAPDH gene in the tested groups. & p < 0.05 vs. CONT group, * p < 0.05 vs. METH group, @ p < 0.05 vs. METH-W group, and ^ p < 0.05 vs. METH-ET group.

Fig 5 illustrates the expression levels of Caspase-1 gene in the cardiac tissue. The ANOVA results revealed a significant difference between the groups (f = 206.48, p < 0.001), indicating differential Caspase-1 gene expression.

Tukey's post hoc tests showed significant upregulation of Caspase-1 expression in the METH group compared to that in the METH+ET-W-ET, METH+ET, SAL, METH+ET-W, METH-ET, CONT and METH-W groups, with mean differences of 2.660, 2.620, 2.610, 2.550, 2.220,2.030 and 1.770, respectively (all p < 0.001). A significant difference was also found between the METH and METH -W groups (mean difference = 3.184, p < 0.001). The METH-W group displayed significantly elevated expression compared to the M + ET-W-ET, METH+ET, SAL, M + ET-W and METH-ET groups, with mean differences of 0.890, 0.580, 0.840.0.780 and 0.450, respectively (all p < 0.001). In the CONT group, significant differences in Caspase-1 expression were observed when compared to the METH+ET-W-ET, METH+ET, SAL and METH -ET groups, with mean differences of 0.630, 0.590, 0.580 and 0.520, respectively (all p < 0.001). Further comparisons between METH-ET and the other treatment groups (METH+ET-W-ET, METH+ET, SAL, and METH+ET-W) indicated significant differences, with mean differences of 0.440, 0.400, 0.390 and 0.330, respectively (all p < 0.001) (Fig 5).

## Discussion

Our findings indicate that METH administration significantly elevates IL-β, IL-18, and Caspase-1 gene expression in rat myocardial tissue. However, endurance training elicits a substantial reduction in the expression of these genes. Furthermore, endurance training (whether applied concurrently with METH administration or subsequent to METH withdrawal) results in a more pronounced decrease in IL-β and caspase-1 gene expression compared to METH withdrawal alone. Moreover, our research demonstrates that the decrease in caspase-1 gene expression following endurance exercise is considerably more pronounced than during the METH withdrawal phase.

Biochemical markers associated with cardiac injury, namely CK-MB and LDH, were investigated, and it was determined that both METH withdrawal and aerobic exercise can significantly lower these cardiac injury markers. Liu et al. (2023) noted substantial increases in CK and LDH levels post METH administration [27]. In their research, Todorovic et al. (2023) discovered that LDH levels were reduced after a four-week period of aerobic exercise. Engaging in physical activity is vital for health maintenance and the prevention of cardiovascular diseases [28,29]. Following injury to cardiac myocytes, metabolic enzymes such as LDH and CK-MB are released into the plasma, with elevated serum levels of these enzymes being key indicators of cardiac injury [7]. Aerobic exercise likely contributes to the reduction of LDH and CK-MB levels by attenuating METH-induced cardiomyocyte damage. Furthermore, it may lessen cardiac tissue injury and signs of cardiotoxicity by enhancing antioxidant enzyme activity and increasing superoxide dismutase (SOD) and catalase (CAT) levels [30].

The reduction in CK-MB and LDH levels may assist in alleviating METH -related toxicity and myocyte injury. Previous studies suggest that exercise training can upregulate heme oxygenase-1 (HO-1) and activate the Nrf2/HO-1 pathway, which helps to reduce oxidative stress and inflammation [31]. Additionally, adaptations to aerobic exercise appear to modulate oxidative stress and mitigate lipid peroxidation, thereby preserving cellular membrane integrity [31].

Current outcomes indicate that METH administration significantly elevates gene expression of inflammatory markers (IL-18, IL-β, and Caspase-1) in rat myocardial tissue. Chronic exposure to METH may affect the expression of cytokines through a variety of signaling pathways and may increase the expression of pro-inflammatory cytokines [10]. METH may cause inflammatory response leading to tissue injury by increasing apoptosis and levels of several pro-inflammatory factors, including TNF-α, IL-6, INF-γ, and nuclear factor-kappa-light chain-enhancer of activated B cells (NF-κb) and NLRP3 [32]. NLRP3 is a crucial member of the PRRs. It combines with ASC and caspase-1 to form a protein known as the NLRP3 inflammasome, which facilitates caspase-1 maturation [33,34]. Mature caspase 1 results in increased maturation and production of pro-inflammatory factors IL-1β and IL-18, leading to inflammatory response leading to tissue injury in various organs [35–37]. IL-18 and IL-1β belong to the interleukin-1 (IL-1) family and are activated by NLRP3. They can to activate intracellular inflammatory signaling cascades and play a critical role in both innate and adaptive inflammatory responses [32]. Cleaved caspase 1 activates GSDMD [38], causing N-terminal fragments to form pores in the cell membrane. This results in the rupture of the plasma membrane and the release of inflammatory cell contents, including IL-1β and mature IL-18, leading to an inflammatory response and pyroptotic cell death [39]. Prior research has demonstrated that METH is capable of activating inflammatory cytokines such as IL-1β, TNF-α, and IL-6 via the NF-κB/STAT3/ERK signaling pathways within cells [40,41]. Furthermore, METH has the potential to induce oxidative damage and auto-oxidation, which leads to the generation of reactive oxygen species (ROS). Additionally, METH interferes with the electron transport chain involved in mitochondrial adenosine triphosphate (ATP) synthesis, causing an accumulation of ROS. The overproduction of ROS results in oxidative stress and subsequent damage. Methamphetamine-induced oxidative stress also results in the production of damage-associated molecular patterns (DAMPs) [42]. In this regard, Saydi et al. (2024) showed that aerobic exercise improves the antioxidant defense system in the lungs of rats after withdrawal from methotrexate [43,44].

Our outcomes also showed that endurance training elicits a substantial reduction in the expression of the IL-1β, IL-18and caspase-1 genes. Furthermore, endurance training (whether applied concurrently with METH administration or after METH withdrawal) results in a more pronounced decrease in IL-1β and caspase-1 gene expression compared to METH withdrawal alone. Notably, exercise has been shown to downregulate key inflammatory mediators, including nodal-like receptor protein-3 (NLRP3) and caspase-1 [45], diminish caspase-1 activity, and lower the secretion of IL-1β and IL-18 [46]. Complementarily, a study by Wang et al. (2021) demonstrated that eight weeks of progressive aerobic training ameliorated certain inflammatory markers, such as TNF-α, IL-6, and IL-1β in individuals undergoing METH withdrawal [47]. Conversely, a study by Li et al. (2022) reported that 21 days of low-intensity aerobic training did not alter specific inflammatory markers, including TNF-α, IL-6, and IL-1β in the cerebral regions of METH-treated rats [48]. It can be inferred that adaptive responses to exercise training confer beneficial effects on reducing inflammation levels. Indeed, enhanced cellular oxygen utilization may play a role in attenuating inflammatory processes [49]. Regular training may decrease sympathetic nervous system activity and augment the production of anti-inflammatory cytokines, thereby mitigating the release of pro-inflammatory mediators [50].

Activation of β-adrenergic receptors may serve as a mechanism through which exercise induces changes in inflammatory mediators. The stimulation of adrenergic receptors in the myocardium enhances the secretion of pro-inflammatory cytokines, and both the density and activity of these receptors may be influenced by exercise training [51]. Numerous pieces of evidence indicate that adrenergic receptors may be involved in the regulation of cytokine release in various chronic inflammatory disease states. These receptors are found on multiple cell types and tissues capable of secreting proinflammatory cytokines, including leukocytes, adipose tissue, and muscle. The precise function of β-adrenergic receptors in the regulation of cytokine release is likely to differ depending on the specific cytokine and the tissue from which it originates. Nonetheless, there are potential connections between inflammatory factors and β-adrenergic receptors across different tissues [52]. Studies indicate that exercise training may lead to a reduction in the density of β-adrenergic receptors in the myocardium, which could consequently decrease the levels of inflammatory markers [51].

## Conclusion

The findings of the present study demonstrate that methamphetamine administration induces myocardial injury, as evidenced by increased serum CK and LDH levels and the upregulation of IL-18, IL-1β, and Caspase-1 gene expression in rat myocardial tissue. In contrast, endurance training significantly attenuated these changes, reducing both myocardial injury markers and the expression of inflammasome-related genes. These findings suggest that endurance training exerts a protective effect against methamphetamine-induced cardiac damage by suppressing inflammatory responses associated with the NLRP3 inflammasome pathway. Overall, endurance exercise may represent a promising non-pharmacological strategy for mitigating methamphetamine-induced myocardial inflammation and injury.

## Limitations

The current study has several limitations. The molecular findings were based primarily on gene expression analysis and were not validated using complementary approaches such as protein expression analyses (e.g., Western blotting or ELISA) or functional assays. Therefore, the proposed mechanisms underlying the protective effects of endurance training should be interpreted with caution. In addition, histopathological evaluation of cardiac tissue (e.g., H&E staining) was not performed, which could have provided further evidence of myocardial injury. Future studies incorporating protein-level analyses, functional assays, and histological assessment are warranted to validate these findings and further clarify the mechanisms involved.

## Supporting information

S1 Data Supplementary data include the raw Ct values, ΔCt, ΔΔCt, and fold-change (2^ − ΔΔCt) calculations for all experimental samples, as well as the individual CK and LDH values used for statistical analyses. (XLSX)
