# Supplementary material for: The impact of endurance training on reducing inflammatory markers in the cardiac tissue of methamphetamine-exposed rats
Source: PLoS One. 2026 Aug 26;21(8):e0356103. doi: 10.1371/journal.pone.0356103 (PMC13514017; doi:10.1371/journal.pone.0356103)
Supplement: Supplementary file 3 [file pone.0356103.s004.docx]

**Response to Reviewer Comments**

**Manuscript Title**: ***The impact of endurance training on reducing inflammatory markers in the cardiac tissue of methamphetamine-exposed rats***

**Manuscript ID**: PONE-D-25-43413R2

Dear Editor and Reviewers,

We sincerely thank you for your thorough review and valuable feedback on our manuscript. Your comments have significantly improved the clarity, ethical transparency, and scientific integrity of our work. Below, we address each of the reviewer comments and outline the changes made to the manuscript to ensure compliance with *PLOS ONE*’s editorial guidelines.

**Response to Reviewers**

- **Question: First, avoid using words that anthropomorphizes like using words like "encourage."**

**Response:** We sincerely thank the reviewer for this valuable editorial suggestion. We have carefully reviewed the entire manuscript and replaced all anthropomorphic, teleological, or subjective terms (including "encourage", "boost", "aids in", "protecting", "bolstering", and similar expressions) with precise, mechanistic, and objective scientific terminology. All modifications have been highlighted in purple in the revised manuscript for ease of review.

- **Question: Second, can you add the name of the supplier of the animal model you used in this manuscript and location of their headquarters.**

**Response:** Rats were purchased from the Pasteur Institute of Iran (Research and Production Complex, Karaj, Iran) at adulthood and maintained under standard laboratory conditions. Rats were obtained at 6 weeks of age from the supplier with an average initial body weight of 200 ± 10 g.

- **Question:Third, add the model name of your qPCR device, and include information on the cycling parameters (denaturation, annealing, extension, cycles, and others like if you used SYBR green, Taqman, or the kit used.**

**Response:** Gene expression was quantified using quantitative polymerase chain reaction (qPCR) with a Corbett 6000 real-time PCR system (Australia) on an Applied Biosystems platform (USA). The thermal cycling program consisted of three distinct stages (Table 1). The initial stage involved a 10‑min incubation at 95 °C to promote DNA strand separation and activate the DNA polymerase enzyme. The second stage comprised a denaturation step at 95 °C for 15 s, followed by primer annealing at 60 °C for 1 min; this cycle was repeated 40 times. The final stage included a melting curve analysis with sequential incubations at 72 °C for 15 s, 60 °C for 30 s, and 95 °C for 18 s.

Real‑time PCR assays were conducted in duplicate using 96‑well plates, with a total reaction volume of 25 µL. Each reaction mixture contained 12.5 µL of SYBR Green master mix with NOROX (Ampliqon, Denmark), 10 pmol (1 µL) of each gene‑specific forward and reverse primer, 20 ng of genomic DNA (5 µL), and nuclease‑free distilled water. SYBR Green I fluorescent dye was used for signal detection.

The optimized temperature protocol in this study after various investigations is as shown in Table 1.

- Table1. The optimized temperature protocol

| Temperature | | Duration of cycle | | Cycles | |  |
| --- | --- | --- | --- | --- | --- | --- |
| 950c | | 15 minutes | | 1 | |  |
| Extention  Denaturation  Annealing | 950c  600c  720c | | 15-30 seconds  30 seconds  30 seconds | | 40 | |

The ΔCT value was calculated using the equation ΔCT = CT(target gene) – CT(GAPDH), with IL‑18, IL‑1β, and caspase‑1 designated as the target genes. Relative gene expression levels were subsequently determined using the 2^–ΔΔCT method. Oligonucleotide primers specific to IL‑18, IL‑1β, caspase‑1, and the housekeeping gene GAPDH were designed based on sequences retrieved from established genomic databases. Real‑time PCR provided the CT and mean CT values for each sample, which were then processed in Excel to generate the corresponding ΔΔCT values. Final gene expression levels were computed according to the 2^–ΔΔCT calculation. The primer sequences used in this study are listed in Table 2.

- **Question: Fourth, there is repetition of how CT and mean CT data and calculation of fold change is determined. Please, avoid repetitions, and remove the text on Table 1. Just refer to the table and change to the title of Table 1 to something like, qPCR primer sequences.**

**Response: Table 2. qPCR primer sequences**

- **Question: Fifth, please provide the name and manufacturer of the LDH kit.**

**Response:** Blood samples were collected from the left ventricle of the mice, centrifuged, and stored at -70°C for analysis. LDH and CK-MB were measured using specialized kits and spectrophotometric techniques. High levels of CK-MB were indicative of myocardial damage18. Serum CK and LDH were measured (PARS AzMUN kit, Tehran, Iran) [26].

- Lactate + NAD + H + Pyruvate + NADH ↔ LDH
- NADH activity leads to the oxidation of the enzyme LDH. The reduction of NAD to NADH in this process is directly proportional and can be assessed photometrically.
- **Question: Sixth, one of the reviewers from the last review suggested changing Table 2 to a figure. That was neither done nor addressed with a reason.**

**Response:** As recommended, Table 2 has now been converted to Figures 1 and 2 in the revised manuscript to improve data presentation and readability.

**Question: Seventh, in the discussion, there is the introduction of SOD and CAT without any meaning to these abbreviations. Authors should first use full name before abbreviations.**

**Response:** superoxide dismutase (SOD) and catalase (CAT)

- **Question: Finally, in the limitation sections, H&E is not used to examined protein analysis. I assume that the authors meant to say immunofluorescence imaging.**

**Response:** There are limitations to the current study, including the lack of analysis of protein expression such as caspase-1, IL-18, and IL-1β by Western blot and ELISA to measure the amount of cardiac tissue proteins. In future research, it is suggested to use methods such as Western blot for protein expression as well as imaging of cardiac tissue using different techniques (such as H&E). A better understanding of the extent of cardiac injury could potentially aid in the treatment of methamphetamine withdrawal patients.
