# Supplementary material for: The impact of endurance training on reducing inflammatory markers in the cardiac tissue of methamphetamine-exposed rats
Source: PLoS One. 2026 Aug 26;21(8):e0356103. doi: 10.1371/journal.pone.0356103 (PMC13514017; doi:10.1371/journal.pone.0356103)
Supplement: Supplementary file 4 [file pone.0356103.s005.docx]

**Response to Reviewer Comments**

**Manuscript Title**: ***The impact of endurance training on reducing inflammatory markers in the cardiac tissue of methamphetamine-exposed rats***

**Manuscript ID**: PONE-D-25-43413R2

Dear Editor and Reviewers,

We sincerely thank you for your thorough review and valuable feedback on our manuscript. Your comments have significantly improved the clarity, ethical transparency, and scientific integrity of our work. Below, we address each of the reviewer comments and outline the changes made to the manuscript to ensure compliance with *PLOS ONE*’s editorial guidelines.

**Response to Reviewers**

**Question:** In addition, although the current results generally support the authors' conclusions, the experimental evidence remains relatively limited, as the molecular findings are primarily based on gene expression analysis without further validation using complementary approaches (e.g., protein-level or functional assays). While I do not consider this issue essential for the current revision, the authors should acknowledge this limitation more explicitly in the Discussion and avoid overstating the mechanistic conclusions.

**Response:** Thank you for your valuable comment. We have revised the manuscript accordingly and explicitly acknowledged this limitation in the Discussion section.

**Question:** In the Methods section of the Abstract (approximately line 34), the methamphetamine dosage is written as "5 mg/ok" instead of "5 mg/kg". Please correct this typographical error.

**Response:** Thank you for your careful observation. The typographical error has been corrected.

**Question:** In the "Measurement of LDH and CK-MB" section (approximately line 199), the term "mice" is used, although the study was conducted in rats. The same issue also appears around line 350. Please revise these to maintain consistency throughout the manuscript.

**Response:** Thank you for your careful observation. The terminology has been corrected to ensure consistency throughout the manuscript.

**Question:** The topmost horizontal comparison bar in Figure 4 (upper right) appears to be unlabeled and does not clearly indicate the corresponding statistical comparison. Please verify whether this is an unintended graphical artifact or provide the appropriate statistical annotation.

**Response:** Thank you for your careful observation. The figure has been revised, and the unnecessary graphical artifact has been removed**.**

**Question:** Please revise the Conclusion to focus on the principal findings of the present study. Information regarding findings from previous studies has already been discussed in the Discussion section and does not need to be reiterated in the Conclusion.

**Response:** Thank you for your valuable comment. The Conclusion has been revised to focus on the principal findings of the present study.
