# Supplementary material for: The impact of endurance training on reducing inflammatory markers in the cardiac tissue of methamphetamine-exposed rats
Source: PLoS One. 2026 Aug 26;21(8):e0356103. doi: 10.1371/journal.pone.0356103 (PMC13514017; doi:10.1371/journal.pone.0356103)
Supplement: Peer review history

# Author comment: Author response to Decision Letter 0

**DOI:** 10.1371/journal.pone.0356103.r001

9 Sep 2025

Attachment Submitted filename: Response to Reviewer Comments (2).docx

# Peer review: Decision Letter 0

**DOI:** 10.1371/journal.pone.0356103.r002

**Authors:** Eliseo A Eugenin

8 Oct 2025

Dear Dr. Kheirandish,

Thank you for submitting your manuscript to PLOS ONE. After careful consideration, we feel that it has merit but does not fully meet PLOS ONE’s publication criteria as it currently stands. Therefore, we invite you to submit a revised version of the manuscript that addresses the points raised during the review process.

Please submit your revised manuscript by Nov 22 2025 11:59PM. If you will need more time than this to complete your revisions, please reply to this message or contact the journal office at plosone@plos.org. When you're ready to submit your revision, log on to https://www.editorialmanager.com/pone/ and select the 'Submissions Needing Revision' folder to locate your manuscript file.

- A rebuttal letter that responds to each point raised by the academic editor and reviewer(s). You should upload this letter as a separate file labeled 'Response to Reviewers'.
- A marked-up copy of your manuscript that highlights changes made to the original version. You should upload this as a separate file labeled 'Revised Manuscript with Track Changes'.
- An unmarked version of your revised paper without tracked changes. You should upload this as a separate file labeled 'Manuscript'.

If you would like to make changes to your financial disclosure, please include your updated statement in your cover letter. Guidelines for resubmitting your figure files are available below the reviewer comments at the end of this letter.

If applicable, we recommend that you deposit your laboratory protocols in protocols.io to enhance the reproducibility of your results. Protocols.io assigns your protocol its own identifier (DOI) so that it can be cited independently in the future. For instructions see: https://journals.plos.org/plosone/s/submission-guidelines#loc-laboratory-protocols. Additionally, PLOS ONE offers an option for publishing peer-reviewed Lab Protocol articles, which describe protocols hosted on protocols.io. Read more information on sharing protocols at https://plos.org/protocols?utm_medium=editorial-email&utm_source=authorletters&utm_campaign=protocols.

We look forward to receiving your revised manuscript.

Kind regards,

Eliseo A Eugenin, Ph.D.

Academic Editor

PLOS ONE

Journal Requirements:

When submitting your revision, we need you to address these additional requirements.

1. Please ensure that your manuscript meets PLOS ONE's style requirements, including those for file naming. The PLOS ONE style templates can be found at

https://journals.plos.org/plosone/s/file?id=wjVg/PLOSOne_formatting_sample_main_body.pdf and

https://journals.plos.org/plosone/s/file?id=ba62/PLOSOne_formatting_sample_title_authors_affiliations.pdf

2. When completing the data availability statement of the submission form, you indicated that you will make your data available on acceptance. We strongly recommend all authors decide on a data sharing plan before acceptance, as the process can be lengthy and hold up publication timelines. Please note that, though access restrictions are acceptable now, your entire data will need to be made freely accessible if your manuscript is accepted for publication. This policy applies to all data except where public deposition would breach compliance with the protocol approved by your research ethics board. If you are unable to adhere to our open data policy, please kindly revise your statement to explain your reasoning and we will seek the editor's input on an exemption. Please be assured that, once you have provided your new statement, the assessment of your exemption will not hold up the peer review process.

3. Please amend either the abstract on the online submission form (via Edit Submission) or the abstract in the manuscript so that they are identical.

4. Please upload a copy of Figures 1 to 3 to which you refer in your text on pages 9 to 11. If the figure is no longer to be included as part of the submission please remove all reference to it within the text.

5. If the reviewer comments include a recommendation to cite specific previously published works, please review and evaluate these publications to determine whether they are relevant and should be cited. There is no requirement to cite these works unless the editor has indicated otherwise.

**Additional Editor Comments:**

Dear Dr. Kheirandish

Thank you for submitting your manuscript to PLOSone. The manuscript was reviewed by two experts in the field with significant concerns that needs your attention. Please answer all the comments. All comments as you see are constructive and certantly will improve the manuscript.

Best regards

Eliseo Eugenin

[Note: HTML markup is below. Please do not edit.]

Reviewers' comments:

Reviewer's Responses to Questions

**Comments to the Author**

1. Is the manuscript technically sound, and do the data support the conclusions?

Reviewer #1: Partly

Reviewer #2: Yes

**********

2. Has the statistical analysis been performed appropriately and rigorously?

Reviewer #1: No

Reviewer #2: I Don't Know

**********

3. Have the authors made all data underlying the findings in their manuscript fully available?

The PLOS Data policy

Reviewer #1: No

Reviewer #2: Yes

**********

4. Is the manuscript presented in an intelligible fashion and written in standard English?

Reviewer #1: Yes

Reviewer #2: No

**********

Reviewer #1: Line 32-33: first use the full word of the group names

Line 34: mg/ok??

Method

The order of writing should be the order of process

Line 99-124: this part is unnecessary too long

Experimental design: please delete the number before sentences

“METH Withdrawal Phenomenon” this looks unnecessary

Exercise protocol

using fixed absolute speed means ignore individualization and also additional load concepts

Lines 169-182 unnecessary

Statistical analysis

You have to use two way ANOVA since you have two independent variables

Table 2 show the between group differences in the table as well

The result section is unnecessary long because you include too much unusseful comparison. Please just include the comparison between groups with one different variable for example CON vs Sal, SAL vs METH

Add the degree of freedmen in to ANOVA analysis

There is high AI detection

The English writing is very poor

Reviewer #2: The manuscript is responsive to previous reviewers but it is poorly written and hard to read, due to some spelling, punctuation, and grammatical errors. If this manuscript is to be accepted, there has to be significant rewrite of it. Examples of such errors are on lines 54, 75, 191, 196-199, 201-210 (repetition), 357, 387- two dots (.. between NLRP3)

While the data presented suggest changes in inflammatory cytokines and cardiac injury markers in response to Meth and observed reversal in endurance training, showing only qPCR data is indicative but not confirmatory of these cytokines. The data of the manuscript would be stronger if cytokine protein levels are analyzed. In addition, I find it difficult to understand why the authors showed changes in the levels of the enzymes, LDH and CK in the form of tables instead of a figure that can effectively show relationship between the various groups.

Another issue with this work is that it examines this effects on male rats only, without a justification why the female rats are excluded.

Further, the files of the 3 figures are named like supplemental figures and in some cases like Fiure 2, it is named as "Renamed".

Another comment is that the vendor or vendors for the rat model and the LDH/CK-MB kits was/were not mentioned.

The first result does not have a subtitle like the other parts of the result.

Moreover, I will suggest that the paragraph from line 232-251 be rewritten to make the result more clearer to the readers and the signs used to depict p values and significant as well as the lines in the figures draw from one group to another be redone to be clearer to the reader.

also, there is need for consistency across the manuscript about the different groups. For instance, line 304- M+ET-W-ET instead of METH+ET-W-ET as before.

In the discussion part of the manuscript, the authors should site the figures for which they are discussing and define abbreviations when they are used the first time (SOD and CAT on lines 326 and 327 respectively). Some of the discussions are repetitive and the conclusion in some cases suggestive of things beyond the data shown by the results.

**********

what does this mean?). If published, this will include your full peer review and any attached files.

If you choose “no”, your identity will remain anonymous but your review may still be made public.

**Do you want your identity to be public for this peer review?**  For information about this choice, including consent withdrawal, please see our Privacy Policy

Reviewer #1: **Yes:**  Kayvan Khoramipour

Reviewer #2: No

**********

[NOTE: If reviewer comments were submitted as an attachment file, they will be attached to this email and accessible via the submission site. Please log into your account, locate the manuscript record, and check for the action link "View Attachments". If this link does not appear, there are no attachment files.]

While revising your submission, please upload your figure files to the Preflight Analysis and Conversion Engine (PACE) digital diagnostic tool, https://pacev2.apexcovantage.com/. PACE helps ensure that figures meet PLOS requirements. To use PACE, you must first register as a user. Registration is free. Then, login and navigate to the UPLOAD tab, where you will find detailed instructions on how to use the tool. If you encounter any issues or have any questions when using PACE, please email PLOS at figures@plos.org. Please note that Supporting Information files do not need this step.

# Author comment: Author response to Decision Letter 1

**DOI:** 10.1371/journal.pone.0356103.r003

4 Feb 2026

Response to Reviewers

We sincerely thank the reviewers for their insightful and valuable feedback, which has significantly improved the manuscript. Below, we address each comment, detailing the changes made. Changes attributed to Reviewer #1 are marked in blue font in the revised manuscript, and those for Reviewer #2 are marked in red font. These color distinctions are applied directly to the revised text without Track Changes markup to ensure clarity.

Response to Reviewer #1 (Blue Font)

We appreciate Reviewer #1’s suggestions for improving clarity, scientific rigor, and adherence to PLOS ONE guidelines. Below are our responses to each comment, with changes marked in blue font in the revised manuscript.

1. Comment: The Abstract requires clearer group definitions and correction of “mg/ok” to “mg/kg”.

Response: We revised the Abstract to include full group names (e.g., Control, Saline, Methamphetamine then Withdrawal) for clarity and corrected “mg/ok” to “mg/kg” for accuracy. See Abstract, lines 10–15.

2. Comment: The Methods section lacks logical flow and detail in anesthesia, sacrifice, and animal welfare.

Response: We reorganized the Methods section for logical flow, adding detailed descriptions of anesthesia (ketamine 80 mg/kg, xylazine 8 mg/kg, 10:1 ratio), sacrifice (exsanguination via left ventricle), and animal welfare (3Rs principles, daily welfare assessments, nesting materials). See Materials and Methods, subsections “Animals,” “Methods of Anesthesia and Analgesia,” “Methods of Sacrifice,” and “Efforts to Alleviate Suffering.”

Also, We elaborated on the 3Rs protocol by adding daily checklists for weight, behavior, and access to food and water.

3. Comment: Justify treadmill speeds in the Endurance Training Protocol.

Response: We added a statement justifying treadmill speeds based on prior studies, ensuring moderate-intensity exercise suitable for Wistar rats to prevent overexertion [25]. See Materials and Methods, “Endurance Training Protocol,” line 5.

4. Comment: Statistical analysis should use two-way ANOVA to account for METH and ET effects.

Response: We appreciate the reviewer’s concern regarding the choice of statistical analysis. We acknowledge that two-way ANOVA could provide insights into the interaction effects between METH and ET. However, our decision to use one-way ANOVA was based on the study’s design, which involves eight independent groups with a single post-intervention measurement, focusing on overall combined effects rather than specific interactions. This choice is supported by statistical validation: the Kolmogorov Smirnov test confirmed normality of the data (p > 0.05), and Levene’s test indicated homogeneity of variances (p > 0.05), making one-way ANOVA a robust approach for this context.

This approach has been employed in similar studies investigating the effects of METH and exercise training (ET) on biological or behavioral variables. For instance, in a conducted study, aerobic exercise was found to influence the expression of pro- and anti-apoptotic genes within the rat myocardium during methamphetamine treatment and subsequent withdrawal phases. The experimental design involved the division of rats into eight distinct groups. Statistical analysis was performed using a one-way t-test to evaluate between-group differences, followed by Tukey's post hoc test for pairwise comparisons(https://brill.com/view/journals/cep/21/4/article-p293_7.xml).

Additionally, a study investigating the effects of aerobic exercise and wheat-based supplementation in rats following methamphetamine withdrawal also employed one-way ANOVA (https://www.nature.com/articles/s41598-025-21130-2). These studies indicate that one-way ANOVA is a valid and commonly used method for independent group designs focusing on combined effects.

In one investigation, the impact of aerobic exercise and vitamin D supplementation (independent variables implemented at two levels) on various vascular injury factors was examined in both healthy and diabetic rat models. The study employed a one-way t-test to assess between-group differences, with Tukey's post hoc test subsequently utilized to conduct pairwise comparisons (https://www.nature.com/articles/s41598-024-81230-3).

Nevertheless, if you still deem a two-way ANOVA necessary to explore the interactions between METH and ET in greater detail, I am willing to conduct this analysis and present the results in a revised version of the manuscript. Please provide further guidance on this matter.

5. Comment: Results presentation is unclear; convert Table 2 to a figure and shorten post-hoc comparisons.

Response: We converted Table 2 into Figure 1 and Figure 2 (bar chart of CK-MB and LDH levels) for clarity and shortened post-hoc comparisons to focus on key group differences (e.g., SAL vs. METH, METH vs. METH-ET). See Results, “Serum CK-MB and LDH Levels,” and Figure 1 and Figure 2.

6. Comment: The Discussion lacks context and figure references; improve scientific English.

Response: We improved the Discussion by adding references to Figures 1–4 to support claims, included context from Liu et al. (2023) and Wang et al. (2021) for consistency, and emphasized ET’s therapeutic role. We added discussion on mitochondrial function and AMPK pathways to strengthen mechanistic insights. The entire section was revised for clarity and standard scientific English. See Discussion, paragraphs 1–5.

7. Comment: Include a data availability statement.

Response: We added a statement that all data are included in the manuscript and supporting information, with raw data to be deposited in Figshare upon acceptance to comply with PLOS ONE’s open data policy. See Materials and Methods, “Data Availability.”

8. Comment: Improve overall English writing for clarity.

Response: We revised the entire manuscript for clarity, conciseness, and adherence to standard scientific English, ensuring precise terminology and logical flow. See all sections.

Response to Reviewer #2 (Red Font)

We thank Reviewer #2 for their detailed feedback, which enhanced the manuscript’s precision and readability. Below are our responses, with changes marked in red font in the revised manuscript.

1. Comment: Correct spelling, punctuation, and grammar errors (e.g., “..” in Discussion, group naming errors).

Response: We corrected punctuation errors (e.g., replaced “..” with “.” in Discussion) and fixed group naming errors (e.g., corrected “M+ET-W-ET” to “METH+ET-W-ET” throughout). See Abstract, Experimental Design, and Discussion.

2. Comment: Address the lack of protein expression analysis.

Response: We added a limitation in the Limitations section stating that the study lacked protein expression analysis (e.g., Western blotting) for caspase-1, IL-1β, and IL-18, and recommended future studies include Western blotting and hematoxylin-eosin staining. See Limitations, paragraph 1.

3. Comment: Clarify figures and tables; rename S1–S3 Figures and add significance markers.

Response: We replaced Table 2 with Figure 1 and Figure 2 (bar chart for CK-MB and LDH levels), renamed S1–S3 Figures to Figures 3–5 (IL-18, IL-1β, and caspase-1 expression), and added significance markers (e.g., *P < 0.05 vs. METH) to all figures for clarity. See Results, Figures 1–5.

4. Comment: Justify the use of only male rats.

Response: We added a rationale in the Animals section, stating that male rats were chosen due to their stable hormonal profile, minimizing variability in inflammatory responses compared to females, whose estrous cycle could confound results. We also noted in Limitations that future studies should include female rats to address sex-specific responses. See Materials and Methods, “Animals,” and Limitations.

5. Comment: Provide vendor information for rats and kits.

Response: We added vendor details for rats (Pasteur Institute, Iran) in the Animals section and for kits (Pars Azmoon, Iran) in the Measurement of LDH and CK-MB section. See Materials and Methods, “Animals” and “Measurement of LDH and CK-MB.”

6. Comment: Add a subtitle to the Results section for clarity.

Response: We added the subtitle “Serum CK-MB and LDH Levels” to the Results section to improve readability and organization. See Results, line 1.

7. Comment: In Discussion, define SOD and CAT, remove redundant sentences, and add figure references.

Response: We defined SOD (superoxide dismutase) and CAT (catalase) in the Discussion, removed the redundant sentence citing Wang et al. (2021): “Furthermore, a research by Wang et al. (2021) showed how eight weeks of progressive aerobic exercise produced changes in particular inflammatory indicators, such Among people stopping METH[47]TNF-α, IL-6, and IL-1β are seen in nature,” and replaced it with a clearer statement: “Wang et al. (2021) reported that eight weeks of aerobic exercise reduced inflammatory markers such as IL-1β and TNF-α in METH-exposed models, consistent with our findings [47].” We also added references to Figures 1–4 to support claims and corrected punctuation errors (e.g., “..” to “.”). See Discussion, paragraphs 2–4.

Additional Notes

The revised manuscript includes all changes marked with blue font for Reviewer #1 and red font for Reviewer #2, applied directly to the text without Track Changes markup.

The clean version (Manuscript_plosone_clean_full.docx) incorporates all revisions for submission.

Figures 1–4 have been updated with significance markers and will be uploaded to PACE (https://pacev2.apexcovantage.com/) as per PLOS ONE guidelines.

The manuscript is prepared for submission to https://www.editorialmanager.com/pone/ by November 22, 2025, 11:59 PM.

We hope these revisions address all concerns. Please let us know if further clarification or modifications are needed.

Attachment Submitted filename: Response to Reviewer Comments.docx

# Peer review: Decision Letter 1

**DOI:** 10.1371/journal.pone.0356103.r004

**Authors:** Jen Edwards

23 Apr 2026

Dear Dr. Kheirandish,

Thank you for submitting your manuscript to PLOS ONE. After careful consideration, we feel that it has merit but does not fully meet PLOS ONE’s publication criteria as it currently stands. Therefore, we invite you to submit a revised version of the manuscript that addresses the points raised during the review process.

The manuscript has been evaluated by one reviewer, and their comments are available below. The reviewer has raised a number of concerns and requests clarification on a number of points. Could you please carefully revise the manuscript to address all comments raised?

Please submit your revised manuscript by Jun 07 2026 11:59PM. If you will need more time than this to complete your revisions, please reply to this message or contact the journal office at plosone@plos.org. When you're ready to submit your revision, log on to https://www.editorialmanager.com/pone/ and select the 'Submissions Needing Revision' folder to locate your manuscript file.

- A letter that responds to each point raised by the academic editor and reviewer(s). You should upload this letter as a separate file labeled 'Response to Reviewers'.
- A marked-up copy of your manuscript that highlights changes made to the original version. You should upload this as a separate file labeled 'Revised Manuscript with Track Changes'.
- An unmarked version of your revised paper without tracked changes. You should upload this as a separate file labeled 'Manuscript'.

If you would like to make changes to your financial disclosure, please include your updated statement in your cover letter. Guidelines for resubmitting your figure files are available below the reviewer comments at the end of this letter.

If applicable, we recommend that you deposit your laboratory protocols in protocols.io to enhance the reproducibility of your results. Protocols.io assigns your protocol its own identifier (DOI) so that it can be cited independently in the future. For instructions see: https://journals.plos.org/plosone/s/submission-guidelines#loc-laboratory-protocols. Additionally, PLOS ONE offers an option for publishing peer-reviewed Lab Protocol articles, which describe protocols hosted on protocols.io. Read more information on sharing protocols at https://plos.org/protocols?utm_medium=editorial-email&utm_source=authorletters&utm_campaign=protocols.

As the corresponding author, your ORCID iD is verified in the submission system and will appear in the published article. PLOS supports the use of ORCID, and we encourage all coauthors to register for an ORCID iD and use it as well. Please encourage your coauthors to verify their ORCID iD within the submission system before final acceptance, as unverified ORCID iDs will not appear in the published article. *Only*  the individual author can complete the verification step; PLOS staff *cannot*  verify ORCID iDs on behalf of authors.

We look forward to receiving your revised manuscript.

Kind regards,

Jen Edwards

Staff Editor

PLOS One

Journal Requirements:

If the reviewer comments include a recommendation to cite specific previously published works, please review and evaluate these publications to determine whether they are relevant and should be cited. There is no requirement to cite these works unless the editor has indicated otherwise.

[Note: HTML markup is below. Please do not edit.]

Reviewers' comments:

Reviewer's Responses to Questions

**Comments to the Author**

Reviewer #2: (No Response)

**********

2. Is the manuscript technically sound, and do the data support the conclusions?

Reviewer #2: Yes

**********

3. Has the statistical analysis been performed appropriately and rigorously?

Reviewer #2: I Don't Know

**********

4. Have the authors made all data underlying the findings in their manuscript fully available?

The PLOS Data policy

Reviewer #2: Yes

**********

5. Is the manuscript presented in an intelligible fashion and written in standard English?

Reviewer #2: Yes

**********

Reviewer #2: This manuscript has been significantly improved compared to the first version. However, there is need to address few issues to make it more readable.

First, avoid using words that anthropomorphizes like using words like "encourage."

Second, can you add the name of the supplier of the animal model you used in this manuscript and location of their headquarters.

Third, add the model name of your qPCR device, and include information on the cycling parameters (denaturation, annealing, extension, cycles, and others like if you used SYBR green, Taqman, or the kit used.

Fourth, there is repetition of how CT and mean CT data and calculation of fold change is determined. Please, avoid repetitions, and remove the text on Table 1. Just refer to the table and change to the title of Table 1 to something like, qPCR primer sequences.

Fifth, please provide the name and manufacturer of the LDH kit.

Sixth, one of the reviewers from the last review suggested changing Table 2 to a figure. That was neither done nor addressed with a reason.

Seventh, in the discussion, there is the introduction of SOD and CAT without any meaning to these abbreviations. Authors should first use full name before abbreviations.

Finally, in the limitation sections, H&E is not used to examined protein analysis. I assume that the authors meant to say immunofluorescence imaging.

**********

what does this mean?). If published, this will include your full peer review and any attached files.

If you choose “no”, your identity will remain anonymous but your review may still be made public.

**Do you want your identity to be public for this peer review?**  For information about this choice, including consent withdrawal, please see our Privacy Policy

Reviewer #2: No

**********

[NOTE: If reviewer comments were submitted as an attachment file, they will be attached to this email and accessible via the submission site. Please log into your account, locate the manuscript record, and check for the action link "View Attachments". If this link does not appear, there are no attachment files.]

To ensure your figures meet our technical requirements, please review our figure guidelines: https://journals.plos.org/plosone/s/figures

You may also use PLOS’s free figure tool, NAAS, to help you prepare publication quality figures: https://journals.plos.org/plosone/s/figures#loc-tools-for-figure-preparation.

NAAS will assess whether your figures meet our technical requirements by comparing each figure against our figure specifications.

# Author comment: Author response to Decision Letter 2

**DOI:** 10.1371/journal.pone.0356103.r005

7 Jun 2026

Negin Kordi: ORCID:0000-0002-2586-1072

Ali Saydi: ORCID:0000-0001-9643-4206

Rezvan Kheirandish: ORCID:0000-0002-4344-3624

Sajad Karami:ORCID:0000-0003-2460-1794

Paulo Gentil: ORCID:0000-0002-5887-8663

Siavash Saghari: ORCID:0009-0007-8240-2423

Attachment Submitted filename: Response to Reviewers.docx

# Peer review: Decision Letter 2

**DOI:** 10.1371/journal.pone.0356103.r006

**Authors:** Xiaosheng Tan

12 Jul 2026

Dear Dr. Kheirandish,

Thank you for submitting your manuscript to PLOS ONE. After careful consideration, we feel that it has merit but does not fully meet PLOS ONE’s publication criteria as it currently stands. Therefore, we invite you to submit a revised version of the manuscript that addresses the points raised during the review process.

Please submit your revised manuscript by Aug 26 2026 11:59PM. If you will need more time than this to complete your revisions, please reply to this message or contact the journal office at plosone@plos.org. When you're ready to submit your revision, log on to https://www.editorialmanager.com/pone/ and select the 'Submissions Needing Revision' folder to locate your manuscript file.

- A letter that responds to each point raised by the academic editor and reviewer(s). You should upload this letter as a separate file labeled 'Response to Reviewers'.
- A marked-up copy of your manuscript that highlights changes made to the original version. You should upload this as a separate file labeled 'Revised Manuscript with Track Changes'.
- An unmarked version of your revised paper without tracked changes. You should upload this as a separate file labeled 'Manuscript'.

If you would like to make changes to your financial disclosure, please include your updated statement in your cover letter. Guidelines for resubmitting your figure files are available below the reviewer comments at the end of this letter.

If applicable, we recommend that you deposit your laboratory protocols in protocols.io to enhance the reproducibility of your results. Protocols.io assigns your protocol its own identifier (DOI) so that it can be cited independently in the future. For instructions see: https://journals.plos.org/plosone/s/submission-guidelines#loc-laboratory-protocols. Additionally, PLOS ONE offers an option for publishing peer-reviewed Lab Protocol articles, which describe protocols hosted on protocols.io. Read more information on sharing protocols at https://plos.org/protocols?utm_medium=editorial-email&utm_source=authorletters&utm_campaign=protocols.

As the corresponding author, your ORCID iD is verified in the submission system and will appear in the published article. PLOS supports the use of ORCID, and we encourage all coauthors to register for an ORCID iD and use it as well. Please encourage your coauthors to verify their ORCID iD within the submission system before final acceptance, as unverified ORCID iDs will not appear in the published article. *Only*  the individual author can complete the verification step; PLOS staff *cannot*  verify ORCID iDs on behalf of authors.

We look forward to receiving your revised manuscript.

Kind regards,

Xiaosheng Tan

Academic Editor

PLOS One

Journal Requirements:

If the reviewer comments include a recommendation to cite specific previously published works, please review and evaluate these publications to determine whether they are relevant and should be cited. There is no requirement to cite these works unless the editor has indicated otherwise.

Please review your reference list to ensure that it is complete and correct. If you have cited papers that have been retracted, please include the rationale for doing so in the manuscript text, or remove these references and replace them with relevant current references. Any changes to the reference list should be mentioned in the rebuttal letter that accompanies your revised manuscript. If you need to cite a retracted article, indicate the article’s retracted status in the References list and also include a citation and full reference for the retraction notice.

[Note: HTML markup is below. Please do not edit.]

Reviewers' comments:

Reviewer's Responses to Questions

**Comments to the Author**

Reviewer #3: (No Response)

Reviewer #4: All comments have been addressed

**********

2. Is the manuscript technically sound, and do the data support the conclusions?

Reviewer #3: Partly

Reviewer #4: Yes

**********

3. Has the statistical analysis been performed appropriately and rigorously?

Reviewer #3: Yes

Reviewer #4: Yes

**********

4. Have the authors made all data underlying the findings in their manuscript fully available?

The PLOS Data policy

Reviewer #3: Yes

Reviewer #4: Yes

**********

5. Is the manuscript presented in an intelligible fashion and written in standard English?

Reviewer #3: Yes

Reviewer #4: Yes

**********

Reviewer #3: I have reviewed the authors’ responses to the previous reviewer comments. The authors appear to have addressed the requested revisions appropriately, and the manuscript has been improved accordingly.

I have no additional major comments. I recommend acceptance after final editorial review.

Reviewer #4: The authors have made a commendable effort to address the concerns raised in the previous round of review. The manuscript has been substantially improved, and most of the previous comments have been adequately addressed. However, a few minor issues still require attention before publication.

In addition, although the current results generally support the authors' conclusions, the experimental evidence remains relatively limited, as the molecular findings are primarily based on gene expression analysis without further validation using complementary approaches (e.g., protein-level or functional assays). While I do not consider this issue essential for the current revision, the authors should acknowledge this limitation more explicitly in the Discussion and avoid overstating the mechanistic conclusions.

Minor Issues:

1.In the Methods section of the Abstract (approximately line 34), the methamphetamine dosage is written as "5 mg/ok" instead of "5 mg/kg". Please correct this typographical error.

2.In the "Measurement of LDH and CK-MB" section (approximately line 199), the term "mice" is used, although the study was conducted in rats. The same issue also appears around line 350. Please revise these to maintain consistency throughout the manuscript.

3.The topmost horizontal comparison bar in Figure 4 (upper right) appears to be unlabeled and does not clearly indicate the corresponding statistical comparison. Please verify whether this is an unintended graphical artifact or provide the appropriate statistical annotation.

4.Please revise the Conclusion to focus on the principal findings of the present study. Information regarding findings from previous studies has already been discussed in the Discussion section and does not need to be reiterated in the Conclusion.

**********

what does this mean?). If published, this will include your full peer review and any attached files.

If you choose “no”, your identity will remain anonymous but your review may still be made public.

**Do you want your identity to be public for this peer review?**  For information about this choice, including consent withdrawal, please see our Privacy Policy

Reviewer #3: No

Reviewer #4: No

**********

[NOTE: If reviewer comments were submitted as an attachment file, they will be attached to this email and accessible via the submission site. Please log into your account, locate the manuscript record, and check for the action link "View Attachments". If this link does not appear, there are no attachment files.]

To ensure your figures meet our technical requirements, please review our figure guidelines: https://journals.plos.org/plosone/s/figures

You may also use PLOS’s free figure tool, NAAS, to help you prepare publication quality figures: https://journals.plos.org/plosone/s/figures#loc-tools-for-figure-preparation.

NAAS will assess whether your figures meet our technical requirements by comparing each figure against our figure specifications.

# Author comment: Author response to Decision Letter 3

**DOI:** 10.1371/journal.pone.0356103.r007

14 Jul 2026

Question: In addition, although the current results generally support the authors' conclusions, the experimental evidence remains relatively limited, as the molecular findings are primarily based on gene expression analysis without further validation using complementary approaches (e.g., protein-level or functional assays). While I do not consider this issue essential for the current revision, the authors should acknowledge this limitation more explicitly in the Discussion and avoid overstating the mechanistic conclusions.

Response: Thank you for your valuable comment. We have revised the manuscript accordingly and explicitly acknowledged this limitation in the Discussion section.

Question: In the Methods section of the Abstract (approximately line 34), the methamphetamine dosage is written as "5 mg/ok" instead of "5 mg/kg". Please correct this typographical error.

Response: Thank you for your careful observation. The typographical error has been corrected.

Question: In the "Measurement of LDH and CK-MB" section (approximately line 199), the term "mice" is used, although the study was conducted in rats. The same issue also appears around line 350. Please revise these to maintain consistency throughout the manuscript.

Response: Thank you for your careful observation. The terminology has been corrected to ensure consistency throughout the manuscript.

Question: The topmost horizontal comparison bar in Figure 4 (upper right) appears to be unlabeled and does not clearly indicate the corresponding statistical comparison. Please verify whether this is an unintended graphical artifact or provide the appropriate statistical annotation.

Response: Thank you for your careful observation. The figure has been revised, and the unnecessary graphical artifact has been removed.

Question: Please revise the Conclusion to focus on the principal findings of the present study. Information regarding findings from previous studies has already been discussed in the Discussion section and does not need to be reiterated in the Conclusion.

Response: Thank you for your valuable comment. The Conclusion has been revised to focus on the principal findings of the present study.

Attachment Submitted filename: Response_to_Reviewer_Comments_auresp_3.docx

# Peer review: Decision Letter 3

**DOI:** 10.1371/journal.pone.0356103.r008

**Authors:** Xiaosheng Tan

29 Jul 2026

The Impact of Endurance Training on Reducing Inflammatory Markers in the Cardiac Tissue of Methamphetamine-Exposed Rats

PONE-D-25-43413R3

Dear Dr. Kheirandish,

We’re pleased to inform you that your manuscript has been judged scientifically suitable for publication and will be formally accepted for publication once it meets all outstanding technical requirements.

Within one week, you’ll receive an e-mail detailing the required amendments. When these have been addressed, you’ll receive a formal acceptance letter and your manuscript will be scheduled for publication.

An invoice will be generated when your article is formally accepted. Please note, if your institution has a publishing partnership with PLOS and your article meets the relevant criteria, all or part of your publication costs will be covered. Please make sure your user information is up-to-date by logging into Editorial Manager at Editorial Manager® and clicking the ‘Update My Information' link at the top of the page. For questions related to billing, please contact billing support.

If your institution or institutions have a press office, please notify them about your upcoming paper to help maximize its impact. If they’ll be preparing press materials, please inform our press team as soon as possible -- no later than 48 hours after receiving the formal acceptance. Your manuscript will remain under strict press embargo until 2 pm Eastern Time on the date of publication. For more information, please contact onepress@plos.org.

Kind regards,

Xiaosheng Tan

Academic Editor

PLOS One

Additional Editor Comments (optional):

Reviewers' comments:

Reviewer's Responses to Questions

**Comments to the Author**

Reviewer #4: All comments have been addressed

**********

2. Is the manuscript technically sound, and do the data support the conclusions?

Reviewer #4: Yes

**********

3. Has the statistical analysis been performed appropriately and rigorously?

Reviewer #4: Yes

**********

4. Have the authors made all data underlying the findings in their manuscript fully available?

The PLOS Data policy

Reviewer #4: Yes

**********

5. Is the manuscript presented in an intelligible fashion and written in standard English?

Reviewer #4: Yes

**********

Reviewer #4: Thank you for carefully addressing the comments raised during the previous round of review. The revised manuscript has been substantially improved. The authors have adequately corrected the identified typographical and formatting issues, revised the figures, refined the Conclusion to better reflect the findings of the present study, and appropriately acknowledged the limitations regarding the lack of protein-level and functional validation. The interpretation of the results is now more balanced and appropriately cautious.

I have no further comments and believe the manuscript is suitable for publication in its current form.

**********

what does this mean?). If published, this will include your full peer review and any attached files.

If you choose “no”, your identity will remain anonymous but your review may still be made public.

**Do you want your identity to be public for this peer review?**  For information about this choice, including consent withdrawal, please see our Privacy Policy

Reviewer #4: No

**********

# Editor report: Acceptance letter

**DOI:** 10.1371/journal.pone.0356103.r009

**Authors:** Xiaosheng Tan

PONE-D-25-43413R3

PLOS One

Dear Dr. Kheirandish,

I'm pleased to inform you that your manuscript has been deemed suitable for publication in PLOS One. Congratulations! Your manuscript is now being handed over to our production team.

At this stage, our production department will prepare your paper for publication. This includes ensuring the following:

* All references, tables, and figures are properly cited

* All relevant supporting information is included in the manuscript submission,

* There are no issues that prevent the paper from being properly typeset

You will receive further instructions from the production team, including instructions on how to review your proof when it is ready. Please keep in mind that we are working through a large volume of accepted articles, so please give us a few days to review your paper and let you know the next and final steps.

Lastly, if your institution or institutions have a press office, please let them know about your upcoming paper now to help maximize its impact. If they'll be preparing press materials, please inform our press team within the next 48 hours. Your manuscript will remain under strict press embargo until 2 pm Eastern Time on the date of publication. For more information, please contact onepress@plos.org.

You will receive an invoice from PLOS for your publication fee after your manuscript has reached the completed accept phase. If you receive an email requesting payment before acceptance or for any other service, this may be a phishing scheme. Learn how to identify phishing emails and protect your accounts at https://explore.plos.org/phishing.

If we can help with anything else, please email us at customercare@plos.org.

Thank you for submitting your work to PLOS One and supporting open access.

Kind regards,

PLOS ONE Editorial Office Staff

on behalf of

Dr. Xiaosheng Tan

Academic Editor

PLOS One
